# Accelerated free breathing ECG triggered contrast enhanced pulmonary vein magnetic resonance angiography using compressed sensing

**DOI:** 10.1186/s12968-014-0091-z

**Published:** 2014-11-22

**Authors:** Sébastien Roujol, Murilo Foppa, Tamer A Basha, Mehmet Akçakaya, Kraig V Kissinger, Beth Goddu, Sophie Berg, Reza Nezafat

**Affiliations:** Department of Medicine (Cardiovascular Division), Beth Israel Deaconess Medical Center and Harvard Medical School, 330 Brookline Ave, Boston, MA 02215 USA

**Keywords:** Magnetic resonance angiography, Pulmonary vein, 3D acquisition, Acceleration techniques, Compressed sensing

## Abstract

**Background:**

To investigate the feasibility of accelerated electrocardiogram (ECG)-triggered contrast enhanced pulmonary vein magnetic resonance angiography (CE-PV MRA) with isotropic spatial resolution using compressed sensing (CS).

**Methods:**

Nineteen patients (59 ± 13 y, 11 M) referred for MR were scanned using the proposed accelerated free breathing ECG-triggered 3D CE-PV MRA sequence (FOV = 340 × 340 × 110 mm^3^, spatial resolution = 1.5 × 1.5 × 1.5 mm^3^, acquisition window = 140 ms at mid diastole and CS acceleration factor = 5) and a conventional first-pass breath-hold non ECG-triggered 3D CE-PV MRA sequence. CS data were reconstructed offline using low-dimensional-structure self-learning and thresholding reconstruction (LOST) CS reconstruction. Quantitative analysis of PV sharpness and subjective qualitative analysis of overall image quality were performed using a 4-point scale (1: poor; 4: excellent).

**Results:**

Quantitative PV sharpness was increased using the proposed approach (0.73 ± 0.09 vs. 0.51 ± 0.07 for the conventional CE-PV MRA protocol, p < 0.001). There were no significant differences in the subjective image quality scores between the techniques (3.32 ± 0.94 vs. 3.53 ± 0.77 using the proposed technique).

**Conclusions:**

CS-accelerated free-breathing ECG-triggered CE-PV MRA allows evaluation of PV anatomy with improved sharpness compared to conventional non-ECG gated first-pass CE-PV MRA. This technique may be a valuable alternative for patients in which the first pass CE-PV MRA fails due to inaccurate first pass timing or inability of the patient to perform a 20–25 seconds breath-hold.

## Background

Atrial fibrillation (AF) is the most common type of cardiac arrhythmia [1]. Triggers arising from pulmonary veins have been shown to be responsible for most AF [2]. Pulmonary vein (PV) isolation (PVI) using catheter ablation [2] is now considered as an accepted treatment of paroxysmal AF [3]. During this procedure, circumferential ablation regions are created at the PV ostia to electrically isolate the PVs. PV anatomies such as the PV ostia size and the number of PVs is generally assessed prior to the PVI procedure using imaging techniques such as multi-detector computed tomography (MDCT) or cardiovascular magnetic resonance (CMR) [4,5]. 3D road maps of the PVs and the left atrium are then generated from these images and are loaded into electro-anatomical mapping system for the guidance of PVI procedures [4,5]. Post PVI imaging is also performed for the detection of rare post-procedural complications such as PV stenosis or damage to the esophagus [6-8].

Both MDCT and CMR are clinically used for left atrium (LA) and PV imaging [5,9-11]. Although MDCT provides improved spatial resolution compared to CMR, it uses iodinated contrast agents and generates ionizing radiation to the patient. Furthermore, a high rate of AF recurrence is observed after PVI procedures, which necessitates redo PVI procedures [9]. Therefore, a non ionizing imaging approach such as CMR would be preferable for PV/LA imaging.

3D contrast-enhanced MR angiography (CE-MRA) is the current standard CMR technique to image both PVs and LA [5,10,12-15]. CE-MRA generally uses a non-ECG triggered spoiled gradient echo (GRE) sequence which is acquired within one prolonged breath-hold during the first pass of a contrast agent. To provide satisfactory contrast in the PVs, the beginning of the CE-MRA acquisition needs to be synchronized with the contrast arrival in the PVs. To this end, a real time sequence is generally acquired during the first pass of the contrast media and stopped at contrast arrival in the right ventricle. Breath-holding instructions are then given to the patients and are immediately followed by the CE-MRA acquisition. The synchronization of the breath-hold initiation with the CE-MRA acquisition and the contrast arrival in the PVs/LA is thus challenging and can fail in some patients, which results in either respiratory motion artifacts and blurring or insufficient contrast in the PVs/LA. 3D non-contrast non-ECG triggered MRA has also been proposed for imaging of the PVs/LA [16-19]. However, this approach is associated with significant loss of both SNR and CNR compared to CE-MRA [16-19] and to motion-induced blurring artifacts which leads to overestimation of the PV size [20].

Furthermore, late gadolinium enhancement (LGE) CMR [21,22] can depict the left atrial wall injury after the radio-frequenty ablation for treatment of AF [23,24]. Post-PVI LGE of LA has also been used as a prognostic tool for identifying patients with AF recurrence [25]. Since 3D MR angiography (MRA) offers a good visualization of the atrial wall, an MRA-driven segmentation can be employed to facilitate the segmentation of the atrial wall and enhanced areas in 3D LA LGE [24-26]. Therefore, this approach requires fusion of MRA and LGE datasets. However, registration of non-ECG gated MRA to ECG-triggered LGE is challenging [24,25]. Therefore, an ECG-triggered 3D MRA could potentially improve the fusion of MRA and LGE datasets.

3D ECG-triggered acquisitions have been proposed in both non contrast MRA [16-19] and CE-MRA [27-30]. Since this approach prolongs the acquisition time, 3D ECG-triggered CE-MRA have been performed within one breath-hold with reduced spatial resolution [30], or under free breathing conditions [27-29] using respiratory navigation techniques [31]. Although high spatial resolution 3D CE-MRA acquisition is desirable for reliable assessment of PV/LA anatomies, it is associated to prolonged scan time which increases the sensitivity to artifact induced by the temporal variation of the contrast agent concentration. Acceleration techniques have been proposed to reduce the scan time using parallel imaging with an acceleration rate of 2 [27,28]. Compressed-sensing (CS) [32,33] is an alternative acceleration technique that enables higher acceleration rates. CS based acceleration has been demonstrated in several applications such as non contrast free breathing PV-MRA using an acceleration factor of 4 to 6 and a retrospective undersampling of the fully-sampled data [19], as well as prospectively in LGE [34,35] using an acceleration factor of 3–4, and contrast enhanced coronary [36] using an acceleration factor of 4.

In this study, we sought to investigate the feasibility of a prospectively-accelerated free breathing, respiratory navigated, ECG-triggered 3D CE-PV MRA sequence using CS in comparison to the clinical gold-standard first-pass breath-hold non-ECG gated 3D CE-MRA.

## Methods

All subjects were scanned using a 1.5 T Philips Achieva (Philips Healthcare, Best, The Netherlands) scanner and a 32-channel cardiac phased array receiver coil. In this health insurance portability and accountability act (HIPAA) compliant study, the imaging protocol was approved by our institutional review board and informed consent was obtained from all participants.

### Study design

**Figure 1:**
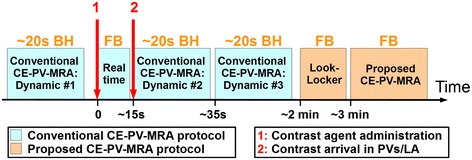
**Study design of the patient study.** The conventional CE-PV-MRA protocol is acquired during the first pass of the contrast agent where each dynamic CE-PV MRA acquisition is performed within one breath-hold (BH). Subsequently, the proposed CE-PV-MRA protocol is started using a Look-Locker sequence and the proposed ECG-triggered free breathing (FB) CE-PV-MRA sequence. The Look-Locker sequence is used to estimate the optimal inversion time (TI) to null myocardial tissue. This optimal TI is then set as inversion time of the proposed ECG-triggered CE-PV-MRA sequence.

Nineteen patients (59 ± 13 years, 11 male) referred for clinical CMR in our center were recruited, including 8 pre-PVI patients and 3 post-PVI patients. All patients were in sinus rhythm at the time of CMR. The study design is shown in Figure 1. Each subject was imaged using the conventional first pass breath-hold 3D CE-PV MRA protocol and the proposed accelerated respiratory navigated ECG-triggered 3D CE-PV MRA protocol. All participants received an injection of 0.1 mmol/kg of gadobenate dimeglumine (MultiHance; Bracco Diagnostic Inc., Princeton, NJ) as a single bolus injection with rate of 2 mL/s.

As part of the clinical protocol, the conventional breath-hold 3D CE-PV MRA sequence was repeated three times, one time before contrast injection for training purpose (Dynamic #1), once during the first pass of the contrast agent (Dynamic #2), and a last time in case of incorrect timing of the first pass acquisition (Dynamic #3). For each of three acquisitions, the subjects were instructed for two maximum capacity respirations followed by a ~20 second end-expiratory breath-hold. The three acquisitions of the conventional breath-hold 3D CE-PV MRA used a gradient recalled echo (GRE) sequence with the following parameters: TR/TE/α =3.2 ms/1.12 ms/40°, FOV =320 × 320 × 90 mm^3^, voxel size = 1.5 × 1.5 × 1.5 mm^3^, SENSE acceleration factor = 2.5 (FH direction), scan duration = 20 s, and frequency, phase, and slice encoding directions = (RL, FH, AP, respectively). To obtain maximum contrast in the PVs and LA, the sequence used a centric profile reordering. In order to synchronize the beginning of the second conventional breath-hold 3D CE-PV MRA acquisition (Dynamic #2) with the first pass of the contrast bolus, a real time sequence was initiated at the time of the contrast agent administration (single bolus injection at 2 mL/s). A single slice was acquired in the coronal orientation using a GRE sequence with the following parameters: TR/TE/α = 3 ms/ 0.87 ms/40°, FOV = 530 × 530 mm^2^, voxel size = 2.1 × 4.1 mm^2^, slice thickness = 80 mm, and temporal resolution = 390 ms. The real time acquisition was stopped upon arrival of the contrast media into the right ventricle and followed by breath-holding instructions and the conventional first pass breath-hold 3D CE-PV MRA (Dynamic #2).

**Figure 2:**
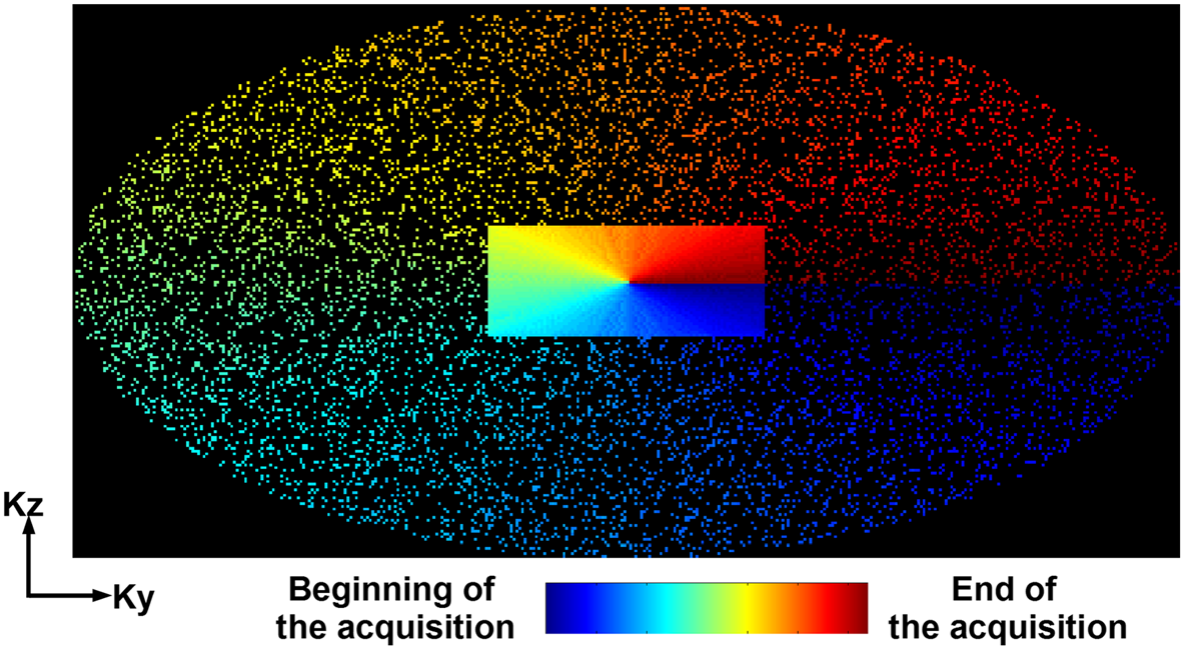
**Illustration of the employed undersampling pattern and k-space profile reordering used for compressed sensing acceleration.**

After completion of the conventional first pass breath-hold 3D CE-PV MRA protocol, a Look Locker sequence [37] was acquired and followed by the proposed accelerated free-breathing ECG-triggered 3D CE-PV MRA, acquired at ~3 minutes after contrast administration. This sequence used an inversion recovery steady-state free precession (SSFP) sequence with the following parameters: TR/TE/α = 4.1 ms/2 ms/90°, FOV = 320 × 320 × 90 mm^3^, voxel size = 1.5 × 1.5 × 1.5 mm^3^, fat saturation using SPIR, and acquisition window = 140 ms, frequency, phase, and slice encoding directions = (FH, RL, AP, respectively). K-space segments were acquired at every RR interval. The inversion time was selected to null myocardial tissue and was estimated from the prior Look Locker sequence. Data were acquired at the mid rest diastolic period which was identified from a cine scan acquired before contrast injection. The sequence was respiratory gated (gating window = 7 mm) and tracked (factor = 0.4 [38,39]) using a pencil beam navigator positioned on the right hemi diaphragm. The position of the pencil beam navigator was slightly shifted away from the dome of the right hemi-diaphragm towards the right hand side of the patient to minimize the intersection between the navigator beam and the PVs and resulting PV inflow artifacts. An acceleration rate of 5 with respect to the elliptical window (acceleration rate of 6.3 with respect to the whole k-space) was employed using a prospective random undersampling pattern [40] (as illustrated in Figure 2). This sampling scheme acquires the full k-space center lines (32 × 19 lines in k_y_-k_z_) and randomly discards outer k-space lines to reach an acceleration factor of 5. A radial re-ordering of the k-space data was used to minimize the k-space jumps and to reduce eddy current artifacts [40]. Note that the same random undersampling pattern was used in all subjects. The average scan time of the proposed sequence was 90 seconds assuming a 100% gating efficiency and 60 beats/minute.

The CS reconstruction was performed using an advanced B_1_-weighted CS reconstruction technique [41] which iteratively alternate between thresholding of the combined coil image using the low-dimensional-structure self-learning and thresholding reconstruction (LOST) [40] and enforcement of data consistency. The reconstruction has been performed offline in the first 2/3 of the patients and then online in the remaining patients using an online in-house reconstruction tool [42]. The average reconstruction time was ~1 h per case.

### Data analysis

**Figure 3:**
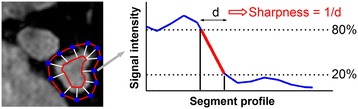
**Protocol used for quantitative sharpness analysis.** The PV sharpness was measured at multiple locations (white segments) and was averaged over all locations. The PV sharpness on a given segment was measured as 1/d where d represents the distance in millimeter required to transition from the 80% threshold (*I*_*min*_ *+ (I*_*max*_ *-I*_*min*_ *) × 0.8*) to the 20% threshold (*I*_*min*_ *+ (I*_*max*_ *-I*_*min*_ *) × 0.2*) of the intensity profile.

Quantitative analysis of the PV sharpness was performed as illustrated in Figure 3. Inner and outer contours of each PV were first manually drawn in the sagittal plane using our in-house platform (MedIACARE) developed in Matlab [43,44]. Each point of the outer contour was then paired with the closest point of the inner contour to generate a virtual segment crossing the PV border. The intensity profile of each segment was then used to measure the PV sharpness as described in [45,46]. The intensity profile along each segment was extracted. Bilinear interpolation was used to increase the sampling density of intensity profiles to 10 points/mm. For each intensity profile, the minimum intensity (*I*_*min*_) and maximum intensity (*I*_*max*_) were measured and two thresholds (*T*_*min*_*, T*_*max*_) were defined as *T*_*min*_ *= I*_*min*_ *+ (I*_*max*_*-I*_*min*_*) × 0.2* and *T*_*max*_ *= I*_*min*_ *+ (I*_*max*_*-I*_*min*_*) × 0.8*. The length (*L*) of the signal transition between these two thresholds was then measured and the sharpness was defined as *1/L*. The sharpness measured over each segments was averaged and used as an overall sharpness measure of the PV.

Subjective qualitative analysis was performed to compare the conventional and the proposed CE-PV MRA sequences. All data were exported in the DICOM format and were loaded in the OsiriX platform (OsiriX 5.7.1; The OsiriX Fondation; Geneva, Swizerland) for image visualization and analysis. Data were visually assessed by an experienced cardiologist (>15 years of experience) who was blinded from the acquisition scheme and patient information. Overall image quality was assessed using a four point scale as: 1: poor image quality (PVs are not visible); 2: fair image quality (some artifacts prevent a clear delineation of all PVs), 3: good image quality (all PV are clearly defined); 4: excellent image quality (all the PVs are clearly defined and sharp).

### Statistical analysis

Paired t-test was used to test the null hypothesis that the difference of quantitative PV sharpness between both approaches is zero. Wilcoxon signed rank test was used to test the null hypothesis that the difference of overall image quality scores between the conventional and the proposed CE-PV MRA sequences was zero. Statistical significance threshold was defined for all tests at p <0.05.

## Results

**Figure 4:**
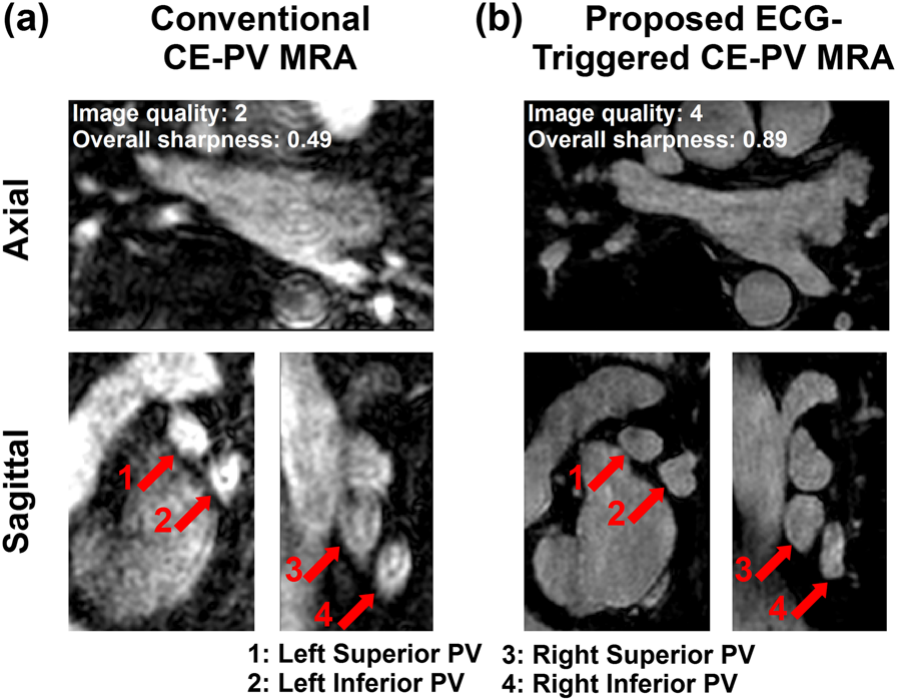
**Conventional (a) and proposed (b) CE-PV MRA obtained in a 63 year-old patient, referred to CMR for assessment of PV/LA anatomy prior to a pulmonary vein isolation procedure.** The conventional CE-PV MRA sequence led to blurring artifacts. PV sharpness and image quality were substantially improved with the ECG-triggered CE-PV MRA sequence.

Figure 4 shows example of PV MRA data acquired in a 63 year-old patient, referred to CMR for assessment of PV/LA anatomy prior to a PVI procedures. Images acquired with the conventional CE-PV MRA protocol (Figure 4a) and the proposed free breathing ECG-triggered CE-PV MRA protocol (Figure 4b) are shown in the axial orientation as well as in two coronal views crossing the left PVs, and the right PVs, respectively. Blurring artifacts and reduced PV sharpness is observed in images acquired with the conventional CE-PV MRA sequence (see arrows). The ECG-triggered CE-PV MRA sequence provided improved PV sharpness (0.89 vs. 0.49) and image quality (4 vs. 2).

**Figure 5:**
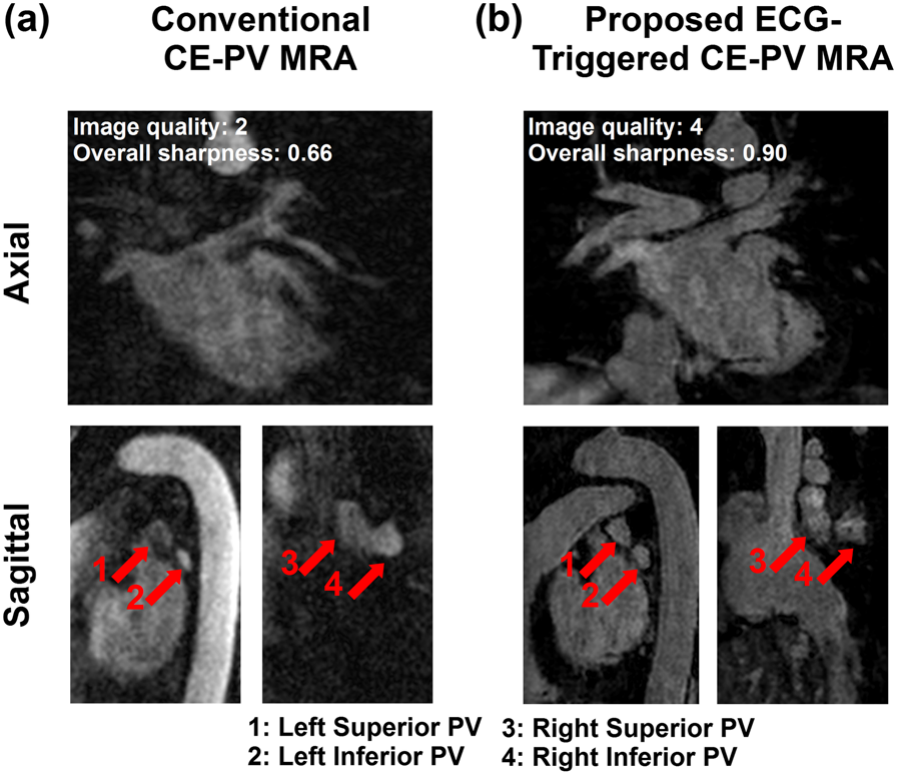
**Conventional (a) and proposed (b) CE-PV MRA sequences acquired in a 48 year-old patient, referred to CMR for assessment of PV/LA anatomy prior to a pulmonary vein isolation procedure.** Low contrast and poor image quality were obtained with the conventional CE-PV MRA sequence due to inaccurate acquisition timing. Improved PV sharpness and image quality were achieved using the proposed ECG-triggered CE-PV MRA sequence.

Figure 5 shows another example of PV MRA data acquired in a 48 year-old patient acquired for assessment of PV/LA anatomy prior to PVI. Due to inaccurate acquisition timing, image acquired with the conventional CE-PV MRA protocol provided low contrast and poor image quality. The proposed ECG-triggered CE-PV MRA sequence resulted in substantial improvement of both PV sharpness (0.90 vs. 0.66) and image quality (4 vs. 2).

Table 1 shows the quantitative analysis of PV sharpness obtained with the conventional CE-PV MRA protocol and the proposed ECG-triggered CE-PV MRA protocol. Over all PVs, the proposed approach provided consistent increased sharpness (0.73 ± 0.09 vs. 0.51 ± 0.07 for the conventional CE-PV MRA protocol, p < 0.001). These differences were also found statistically significant for all individual PV (p < 0.002). There was no statistical difference between the sharpness of the right PVs and left PVs using the conventional approach (0.50 ± 0.08 vs. 0.53 ± 0.09, p = 0.17). However, higher sharpness was measured in the left PVs when compared to the right PVs using the proposed approach (0.70 ± 0.09 vs. 0.75 ± 0.10, p = 0.04).

**Table 1:** **PV sharpness obtained with the conventional first pass non ECG-triggered CE-PV MRA (conventional CE-PV MRA) and the proposed free breathing ECG-triggered CE-PV MRA (proposed CE-PV MRA)**

	**Sharpness (mm** ^**−1**^ **)**		
	**Conventional CE-PV MRA**	**Proposed CE-PV MRA**	**P value**
**Left superior PV**	0.51 ± 0.12	0.75 ± 0.14	P < 0.001
**Left inferior PV**	0.54 ± 0.10	0.75 ± 0.10	P < 0.001
**Right superior PV**	0.48 ± 0.1	0.72 ± 0.10	P < 0.001
**Right inferior PV**	0.52 ± 0.07	0.69 ± 0.10	P = 0.002
**All PVs**	0.51 ± 0.07	0.73 ± 0.09	P < 0.001

The proposed CE-PV MRA protocol led to increased sharpness of all PVs.

Table 2 shows the qualitative analysis of overall image quality. With the conventional CE-PV MRA protocol, 1 dataset received a score of 1, 3 datasets received a score of 2, 4 datasets received a score of 3, and 11 datasets received a score of 4 (15 datasets (79%) received an overall image quality score ≥3 (good or excellent)). With the proposed ECG-triggered CE-PV MRA protocol, none of dataset received a score of 1, 3 datasets received a score of 2, 3 datasets received a score of 3, and 13 datasets received a score of 4 (16 datasets (84%) received an overall image quality score ≥3 (good or excellent)). In the four datasets which received an overall quality score ≤2 using the conventional CE-PV MRA protocol, the proposed ECG-triggered CE-PV MRA protocol provided an overall image quality score of 4. In the three datasets which received an overall quality score ≤2 using the proposed CE-PV MRA protocol, the conventional ECG-triggered CE-PV MRA protocol provided an overall image quality score of 4. Furthermore, at least one dataset (among the two sequences) received an image quality score of 4 in all subjects. The image quality of the proposed technique was superior, equal, and inferior than the conventional CE-PV MRA technique in 8, 6, and 5 cases, respectively. Overall, there were no statistically significant differences in image quality scores between the two techniques (3.5 ± 0.8 vs. 3.3 ± 0.9 using the conventional CE-PV MRA protocol, p > 0.05). The acquisition time of the proposed sequence was 212 ± 65 s which corresponded to a gating efficiency of 44 ± 12% with a heart rate of 62 ± 10 bpm.

**Table 2:** **Qualitative analysis of overall image quality**

	**Conventional CE-PV-MRA**	**Proposed CE-PV-MRA**	**P value**
**Overall image quality**	3.3 ± 0.9	3.5 ± 0.8	0.63

Although differences between both approaches did not reach statistical significance, there was a tendency towards increased overall image quality using the proposed CE-PV-MRA protocol.

## Discussion

In this study, we demonstrated the feasibility of a five-time accelerated free-breathing ECG-triggered CE-PV MRA acquisition using CS. The method was successfully validated in a patient cohort. This approach has higher acceleration rate than previous free-breathing ECG-triggered CE-PV MRA studies [27-29]. Increased PV sharpness was obtained with the proposed approach when compared to a conventional first pass non ECG-triggered CE-PV MRA protocol. There were no differences in subjective image quality scores between the two techniques.

In this study, we used CS as acceleration technique. Other acceleration approaches such as parallel imaging have been previously used for ECG-triggered CE-PV MRA [27,28]. These approaches used lower acceleration factor of 2. Although parallel imaging has been used with higher rate for breath-hold non-contrast thoracic MRA [47,48], its feasibility for free breathing CE-PV MRA has not been shown yet. Furthermore, due to noise penalty induced by the g-factor maps, parallel imaging has been shown to result in higher noise level than compressed sensing [41]. Our CS reconstruction used a B_1_-weighted technique [41] which exploits the sparsity of similar voxel blocks in a 3D volume. Other CS reconstructions have been proposed based on total variation (TV) [32,49], wavelet domain [50], or a combination of TV and wavelet domains [33,51]. However, these techniques were not evaluated in this study.

A radial k-space profile reordering was used for the acquisition of the randomly under-sampled k-space. Although this technique minimizes jumps during each segment acquisition, it increases the sensitivity to k-space weighting induced artifacts since the k-space center lines are acquired over the entire acquisition. Advanced profile reordering techniques which first acquire all k-space center lines over the first heart beats [52,35] may reduce the sensitivity of our approach to k-space weighting induced artifacts and should be investigated in future work.

A CS acceleration factor of 5 was used in this study after performing a pilot study to evaluate overall image quality by changing the acceleration rate from 3 to 6. Several factors could impact the maximum acceleration factor such as baseline SNR, imaging resolution and hardware. In our previous study using 5 channel cardiac coil [34], we found that an acceleration rate of 3 provide excellent image quality. Using 32-channel coil allows us to perform a B_1_-weighted LOST reconstruction that enabled to acquire data with acceleration as high as 6 [41]. Furthermore, imaging sequence will impact the maximum achievable acceleration rate. For example LGE has lower baseline SNR because of use of an inversion pulse, therefore, lower acceleration factor can be used, while non-contrast coronary CMR which has better SNR can be acquired with higher acceleration.

In this study, we used 0.1 mmol/kg of gadobenate dimeglumine and data acquisition was performed in the following ~3 min. There will be some changes in the contrast media in the blood during the acquisition time, which will result in signal falloff and extra weighting on k-space. We have previously studied the changes in T_1_ after gadobenate dimeglumine contrast injection [53] and our data demonstrated that the changes are relatively small for the period of time required for the proposed sequence. Nevertheless, to mitigate the effect of the contrast wash-out, this sequence was designed to minimize the overall scan time using a high acceleration factor combined with a data acquisition at every RR interval. The use of alternate R-wave acquisition would reduce the impact of RR variations on the sequence but would increase the overall scan time by a factor of two. In this study, we decided to privilege the minimization of the contrast wash out effect at the cost of increased sensitivity to RR variations.

The inversion time was adjusted for each patient using a prior Look Locker acquisition and the selected inversion time was kept constant throughout the entire duration of the proposed ECG-triggered CE-PV MRA scan. Although the optimal inversion time variation is higher during the first minutes following the contrast administration [53], a satisfactory nulling of the myocardial signal was achieved in most patients. However, this effect could lead to artifacts in patients with very low gating efficiency which would result in prolonged acquisitions. Advanced k-space profile reordering, as previously discussed, may decrease the sensitivity of the method to the time variation of the optimal inversion time. Adaptive adjustment of the inversion time during the acquisition as initially proposed for late gadolinium enhancement (LGE) imaging [54] could be a valuable option for these patients.

In this study, the respiratory navigator was slightly shifted away from the dome of the right hemi-diaphragm to minimize PV inflow artifacts. Nevertheless, the efficiency of this strategy could be decreased in the presence of certain orientation/anatomies of the heart, as suggested by the slightly lower sharpness measurements obtained in the right PVs. Several approaches have been recently proposed to reduce PV inflow artifacts by modifying the timing of the navigator restore pulse [55] or the timing of the actual navigator signal acquisition [39]. However, these methods were not used in our study.

The proposed sequence is also independent of the breath-hold ability of the patient and does not require any specific breathing pattern. This approach is thus well suitable and compatible with a contrast-enhanced CMR clinical exam. Furthermore, conventional first pass CE-PV MRA may fail with inaccurate first pass timing prediction or in patients with inability to sustain a 20–25 seconds breath-hold. Therefore, our approach represents a valuable CE- alternative PV MRA protocol which could be immediately run when the conventional first pass CE-PV MRA protocol fails.

The proposed sequence has been designed for patients being imaged in sinus rhythm since the majority of patients with paroxysmal AF are most of the time in sinus rhythm during MR imaging. Since all patients in this study were in sinus rhythm at the time of imaging, the impact of AF event on image quality was not quantified. However, the presence of AF event during imaging is expected to create substantial motion/blurring artifacts using the proposed sequence. Further studies are warranted to evaluate this approach in patients being imaged during AF events.

In the methodology used for sharpness quantification, it is difficult to ensure that all lines joining the inner and outer contours were perpendicular to the PV border. Therefore, this may have led to reduced PV sharpness measurements. However, since the same methodology has been used for the analysis of all the data, the potential bias induced between both techniques should have been kept to the minimum.

Despite the PV sharpness improvement achieved using the proposed approach, no statistical difference was obtained in term of qualitative scores between both sequences. This could be explained by several factors. First the narrow score scale combined with the small patient cohort may have limited the assessment of differences between the two approaches. Furthermore, despite the overall higher level of blurring artifact in conventional images, the reader still felt confident in assessing the PVs anatomy in most cases, therefore reducing the spread of the subjective scores.

This study has several limitations. Our patient population was small and could have limited the assessment of differences for qualitative metrics. Both signal to noise ratio (SNR) and contrast to noise ratio (CNR) were not examined in our study due to non-linear LOST reconstruction. The qualitative analysis was only performed by one cardiologist. However, based on his extensive experience in reading of clinical CMR images including PV MRA images, the potential scoring approximation should have been kept to the minimum. Finally, the benefit of the proposed approach for the planning and guidance of PVI procedures as well as the detection of potential post-procedural complications was not evaluated.

## Conclusions

CS-accelerated free-breathing ECG-triggered 3D CE-PV MRA allows evaluation of PV anatomy with improved sharpness compared to conventional non-ECG gated first-pass 3D CE-PV MRA. This technique may be a valuable alternative for patients in which the first pass CE-PV MRA fails due to inaccurate first pass timing or inability of the patient to perform a 20–25 seconds breath-hold.

## Abbrevations

AF: Atrial fibrillation
CE: Contrast enhanced
CNR: Contrast to noise ratio
CMR: Cardiovascular magnetic resonance
CS: Compressed sensing
ECG: Electrocardiograms
FOV: Field of view
GRE: Gradient recalled echo
HIPAA: Health insurance portability and accountability act
LA: Left atrium
LGE: Late gadolinium enhancement
LOST: Low-dimensional-structure self-learning and thresholding reconstruction
MDCT: Multi-detector computed tomography
MRA: Magnetic resonance angiography
CMR: Cardiovascular magnetic resonance
PV: Pulmonary vein
PVI: Pulmonary vein isolation
SENSE: Sensitivity encoding
SNR: Signal to noise ratio
SSFP: Steady-state free precession
TE: Time of echo
TR: Time of repetition
TI: Inversion time
